# Multimodal ultrasound for preoperative evaluation of dermatofibrosarcoma protuberans: a series of 40 cases

**DOI:** 10.1186/s12885-022-10211-4

**Published:** 2022-11-05

**Authors:** Xia Gong, Jia Li, Angang Ding, Jun Chen, Xiaofeng Tao, Ping Xiong, Yamin Rao, Yang Liu, Qilin Sun

**Affiliations:** 1 Department of Radiology, Shanghai Ninth People’s Hospital, School of Medicine, Shanghai Jiaotong University, Shanghai, People’s Republic of China; 2 Department of Ultrasound, Shanghai Ninth People’s Hospital, School of Medicine, Shanghai Jiaotong University, Shanghai, People’s Republic of China; 3 Department of Dermatology and Dermatologic Surgery, Shanghai Ninth People’s Hospital, School of Medicine, Shanghai Jiaotong University, Shanghai, People’s Republic of China; 4 Department of Pathology, Shanghai Ninth People’s Hospital, School of Medicine, Shanghai Jiaotong University, Shanghai, People’s Republic of China

**Keywords:** Dermatofibrosarcoma protuberans, Ultrasound, Elastography, Contrast- enhanced ultrasound

## Abstract

**Background:**

Dermatofibrosarcoma protuberans (DFSP) is a rare, low to intermediate-grade sarcoma, which needs imaging examination. Small series of ultrasound findings in DFSP have been published; however, the usefulness of elastography and contrast-enhanced ultrasound (CEUS) in DFSP has not been studied. We aim to study multimodal ultrasound findings and report the correlation between imagings and tiny extension in DFSP for preoperative evaluation.

**Methods:**

Two-D ultrasound, 3-D color ultrasound, elastography, and CEUS findings were retrospectively evaluated. Forty histopathologically confirmed DFSPs were studied.

**Results:**

On 2-D ultrasound, 26(65%) appeared as mostly hypoechoic lesions with occasional hyperechoic dots within the tumor matrix and lobulated lateral borders. Eight (20%) lesions were multilayered. Ninety-five percent of lesions showed increased vascularity. On 3-D ultrasound, DFSPs showed branch-shaped, striped, and wrapped color patterns. Power Doppler showed mainly artery of a moderate arterial peak systolic blood flow and low resistance index. DFSP is hard on elastography. On CEUS, DFSPs showed a long peak time, low peak and a small amount of perfusion around the tumor, 73.7% (14/19) of lesions showed a heterogeneous contrast enhancement and 89.5% (17/19) of lesions showed hyper-enhancement. CEUS showed better concordance than US with histology on the maximum diameter and depth (*P* < 0.05).

**Conclusions:**

Multimodal ultrasound showed significant characteristics in DFSP, which would improve diagnostic accuracy. CEUS could be an effective tool to determine tiny tumor extension.

## Introduction

Dermatofibrosarcoma protuberans (DFSP) is a low- to intermediate-grade sarcoma, which has a high local recurrence because of its pseudopodia like growth pattern [1]. The tumor tends to invade deep surrounding local structures such as subcutaneous tissue, muscle and, exceptionally, bone [2]. DFSP is usually ignored by patients due to its slow growth pattern [3]. The skin lesion is usually slightly hyper-pigmented with a reddish to bluish color and nodular in appearance [4]; it preferentially affects the trunk of young and middle-aged adults [5–7]. Histopathologically, DFSP is characterized by a uniform spindle cell arrangement, typically with a storiform pattern and CD34 immunoreactivity [1].

In addition to a thorough history and physical examination, diagnosis of suspected DFSP ideally requires a generous biopsy for a pathologic diagnosis. Ultrasound (US) examinations are quick, accessible, and inexpensive, which is the first-line modality for evaluating soft tissue tumors. Some case reports have documented US findings in patients with DFSP [8–19]. Recent study reported grayscale US findings in 30 DFSP cases [18]; one study compared 23 primary and 35 recurrent DFSP cases with grayscale and color Doppler US features [19]; another study reported one case with contrast-enhanced ultrasound (CEUS) examination, and found that CEUS improved precision of resection [13].

In this study, we focused on multimodal ultrasound findings in 40 primary cases, including the usefulness of 2-D US, 3-D color US, elastography and CEUS in DFSP, and the correlation between imagings and tiny extension in DFSP for preoperative evaluation.

## Materials and methods

### Patients

The authors’ institutional review board approved the collection and analysis of retrospective data, while the study protocol was approved by the ethics committee. Preoperative sonography examination data for 40 cases between January 2015 and June 2021 were retrieved for analysis. Diagnoses were confirmed by pathology results. Items reviewed in the medical records of each patient included age, sex, clinical presentation, onset, and location. The follow-up interval for each patient ranged from 3 months to 3 years (mean, 1.5 years).

### US examination

Sonography was performed before the treatment using the GE Voluson E8 instrument (GE Healthcare, Austria) and MyLab Class C (Esaote, Italy), with a broadband (9–14 MHz) linear transducer. The scanner was equipped with a sonoelastographic unit for measuring the lesion elasticity, which is represented by colors. The E8 was equipped with a 3-D probe for superficial lesions. Imaging assessment of all patients was performed by two ultrasound specialists with 8 years of experience.

On 2-D ultrasound images, the size, depth (when the use of extended field of view ultrasound was not adequate to measure lesion size), echotexture (homogeneous or heterogeneous), echogenicity compared with adjacent muscle (hyperechoic, isoechoic, hypoechoic, mixed echo with hyper- and hypo- echo), and margin (well-defined or ill-defined) were evaluated. Vessel density was estimated by counting the number of vessels per square centimeter outlined on color Doppler imaging [20]. Color flow pattern was divided into three groups: no, poor, and rich. The vascularity was divided into peripheral, intralesional, and both. Arteriovenous spectrum, blood flow velocity (arterial and venous) were determined using pulse Doppler ultrasound. The resistance index (RI) was calculated using the following formula:

$$\textrm{RI}=\left(\textrm{PSV}-\textrm{EDV}\right)/\textrm{PSV},$$

where PSV represents peak systolic velocity and EDV is end-diastolic velocity.

3-D color flow pattern was divided into three groups―striped, branch-shaped, and wrapped blood flow. Sonoelastographic images were analyzed using a standardized color scale, with blue indicating regions with low elasticity (stiffer tissue areas) and red indicating regions with high elasticity (soft tissue areas). Sonoelastographic images was performed using the scoring system proposed by investigators from Tsukuba University (Tsubuka, Japan) [21], which distinguishes five types of lesions: 1 = lesions with deformability similar to surrounding tissue (e.g., cysts); 2 = lesions with inhomogeneous deformability and solid components (e.g., benign tumor); 3 = lesions with an elastic periphery and stiff core (unclear signs); 4 = rigid lesions (suspected cancer); and 5 = rigidity of the entire lesion and surrounding tissue (infiltrating cancer). The elastic images were recorded by two scorers with 8 years of experience, and one elastic score per lesion was made by the two together. If necessary, a third opinion of a senior radiologist would be included to draw the final conclusion.

CEUS was performed using an ultrasound system (Esaote MyLab Twice) equipped with a high-energy linear probe of 7–12 MHz, which allows working in fundamental B-mode and in power Doppler mode. As a first step, the suspicious region was examined using conventional ultrasound. Then, DFSP lesions were examined with CEUS. A suspension of the contrast agent was obtained by adding 5 mL of physiological saline to SonoVue (Bracco SpA, Milan, Italy). A contrast bolus of 2.5 mL was injected into the median cubital vein, followed by a wash with 5 mL of saline. Then, the DICOM dynamic data were stored. Each contrast imaging acquisition lasted for at least two continuous minutes, and the process was performed by the software QontraXt.

On CEUS, the DFSPs were evaluated for the following characteristics: depth, the maximum diameter; homogeneity of enhancement was classified as homogeneous or heterogeneous; enhanced intensity (the soft tissue around the lesion as a reference) was classified as iso-enhancement, hyper- enhancement, hypo-enhancement, or no enhancement; peak time (TP) of contrast agent; peak.

### Depth comparison of histology, US and CEUS examination

DFSP depth and the maximum diameter on histology was measured to analyze the difference with US and CEUS. Depth and the maximum diameter on CEUS were recorded on peak time.

### Data analysis

Statistical evaluation was performed using SPSS version 17.0 (IBM Corporation, Armonk, NY, USA). A normality test was conducted to check whether variables had normal distribution. The depth and the maximum diameter comparison analysis were performed using Student’s t-test. *P* < 0.05 was considered statistically significant.

## Results

### Patient characteristics

Forty patients (20 men and 20 women) with a mean age of 38.7 years (range, 15–72 years) were studied. The most common location for DFSPs was the trunk (31/40 cases, 77.5%), followed by the upper or lower limbs (5 cases, 12.5%) and the head and neck (4 cases, 10%). Red nodules on the skin (62.5%; 25 of 40) were found more frequently in the DFSP group, followed by the multinodular (12.5%; 5 of 40), reddish plague (12.5%; 5 of 40), atrophic plague (10%; 4 of 40), and other palpable (2.5%; 1of 40). All lesions were treated with Mohs micrographic surgery, performed within a week of ultrasound.

### US findings

In the DFSP group, 29 cases were measured according to size and depth using ultrasound; the other 11 cases were too large and measured according to the maximum depth. Most of the tumors (26/40, 65%) appeared as mostly hypoechoic lesions with occasional hyperechoic dots within the tumor matrix and lobulated lateral borders. Seven (17.5%) lesions appeared as mostly hyperechoic lesions with occasional hypoechoic bands or hypoechoic areas in the dermis. The remaining 7 tumors (17.5%) had a mixed pattern (hyperechoic and hypoechoic areas); among them, 5 lesions had hyperechoic rings. One case had 3 variations of echo as hypoechoic, hypoechoic with some projections and posterior hyperechoic area, and mixed. The maximum nodule in this case presented as hypoechoic, which was measured by depth and evaluated in our data. Thirty-one (77.5%) lesions were subcutaneous, extending from the skin to the deep fascia over the muscle; 7 (17.5%) lesions were subcutaneous, extending from the skin to the deep fascia and muscle; 1 (2.5%) lesion was subcutaneous, extending from the skin to bone; and 1 (2.5%) lesion was deeply subcutaneous, involving no skin.

On color Doppler, 52.5% (21/40) of lesions were rich; 42.5% (17/40) of lesions were poor. For 21 rich-vascular lesions, 33.3% (7/21) were hypoechoic, 33.3% (7/21) were mixed, 19.1% (4/21) were hypoechoic with projections, and 14.3% (3/21) were hyperechoic with a few hypoechoic bands. In terms of vascular pattern, nearly half (18/40, 45%) of all lesions were intralesional.

**Fig. 1:**
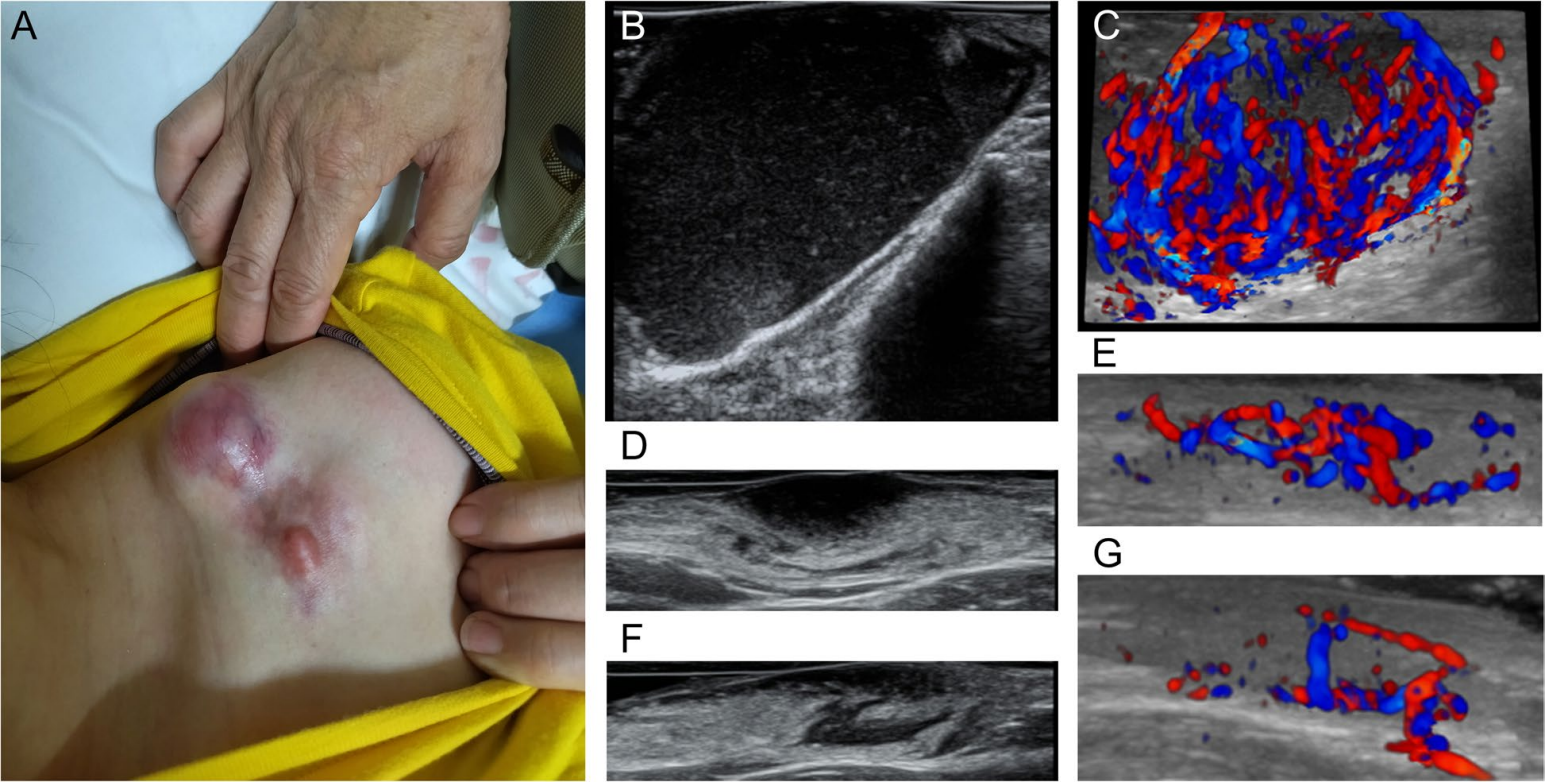
**A** DFSP with three nodules in the chest wall of a 22-year-old woman. **B** Transverse ultrasonogram (No. 1) revealing a well-defined, hypoechoic, homogeneous, and subcutaneous lesion. **C** 3-D color Doppler ultrasonogram revealing wrapped blood flow signal. **D** Transverse ultrasonogram (No. 2) revealing an ill-defined, hypoechoic with finger-like projections and posterior hyperechoic area, heterogeneous, and subcutaneous lesion. **E** 3-D color Doppler ultrasonogram revealing branch-shaped blood flow signal. **F** Transverse ultrasonogram (No. 3) revealing an ill-defined, mixed, heterogeneous, and subcutaneous lesion. **G** 3-D color Doppler ultrasonogram revealing branch-shaped blood flow signal

**Fig. 2:**
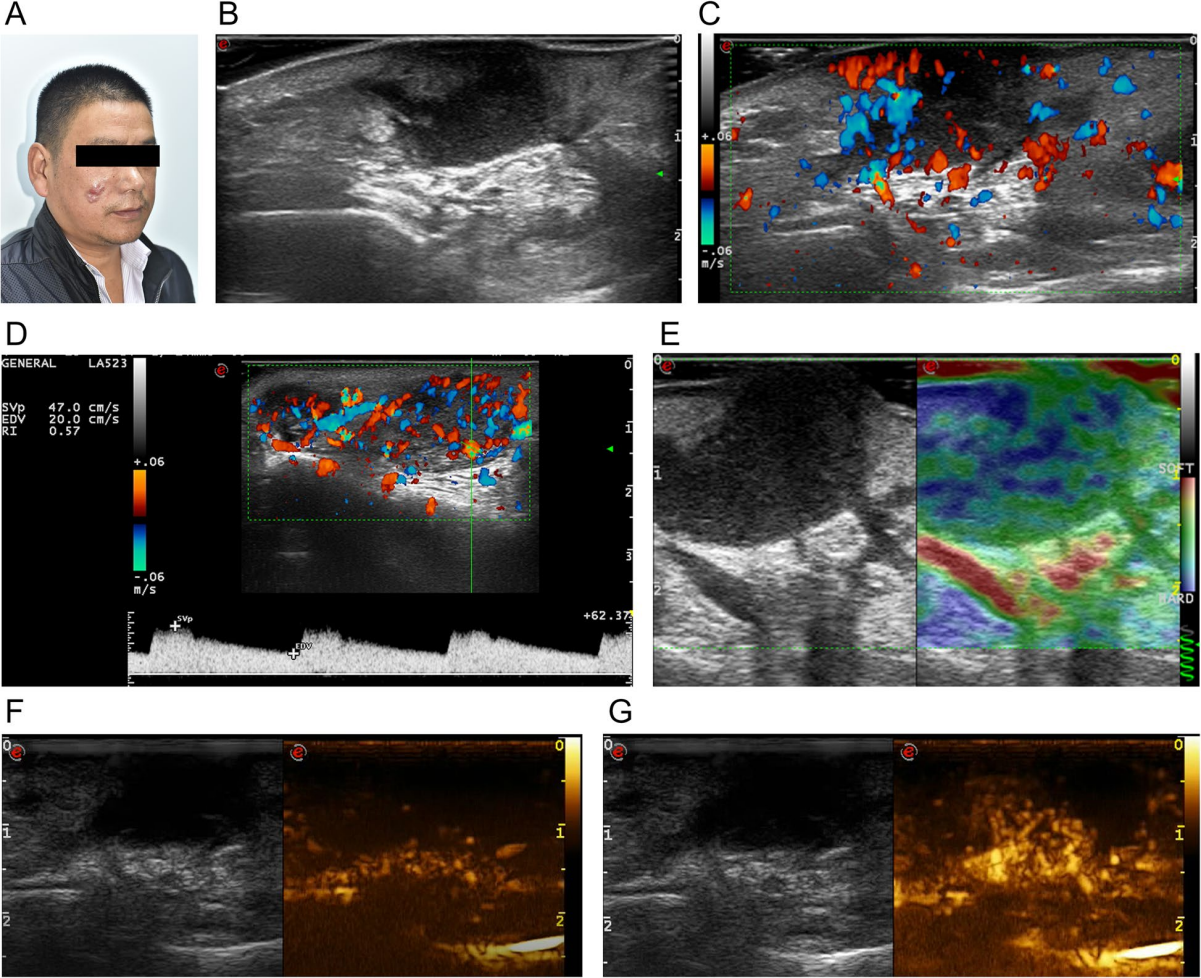
**A** DFSP with three nodules in the right face of a 48-year-old man. **B** Transverse ultrasonogram revealing an ill-defined, hypoechoic with finger-like projections and posterior hyperechoic area, heterogeneous, and subcutaneous lesion extending from the skin to bone. **C** Color Doppler ultrasonogram revealing intralesional and peripheral vascularity and rich vascularization with vascular densities reaching 4.5/cm^2^. **D** Power Doppler ultrasound revealed artery with peak systolic blood flow velocity of 47.0 cm/s and resistance index of 0.57. **E** Elastography showing an elasticity score of 3. **F** Contrast-enhanced ultrasound revealed the trend that contrast agent enters (the 26th second after the injection of contrast agent) the lesion from the bottom to the center. **G** Contrast-enhanced ultrasound revealed heterogeneous hyper-enhancement at peak (the 50th second after the injection of contrast agent), with the maximum diameter and depth of 4.3 cm and 2.1 cm respectively

**Fig. 3:**
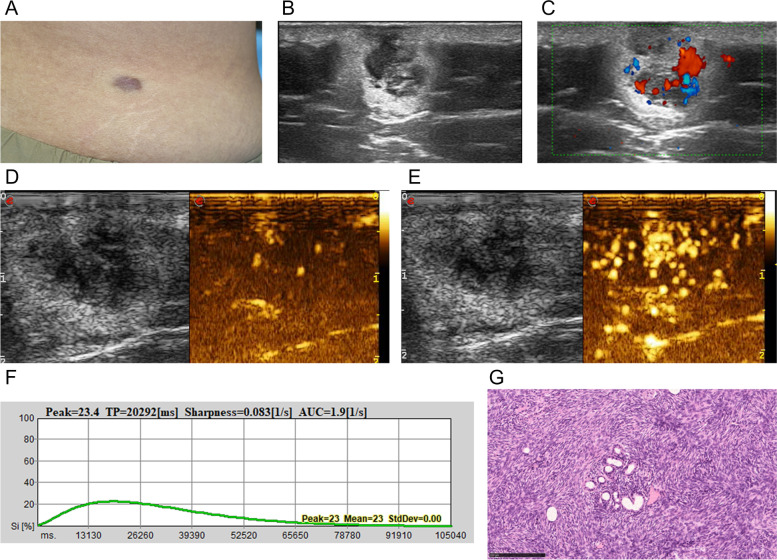
**A**, DFSP with reddish plague in the right waist of a 36-year-old woman. **B** Transverse ultrasonogram revealing a well-defined, mixed with a hyperechoic ring, heterogeneous, and subcutaneous lesion. **C** Color Doppler ultrasonogram revealing intralesional and peripheral vascularity and rich vascularization with vascular densities reaching 3.5/cm^2^. **D** Contrast-enhanced ultrasound revealed the trend that contrast agent enters the lesion from the bottom to the center. **E** Contrast-enhanced ultrasound revealed heterogeneous hyper-enhancement at peak (the 20th second), with the maximum diameter and depth of 2.4 cm and 1.6 cm respectively. **F** Time intensity curve revealed peak of 23.4. **G** The hypoechoic area correlated with a storiform growth pattern on histology (hematoxylin-eosin × 40)

**Fig. 4:**
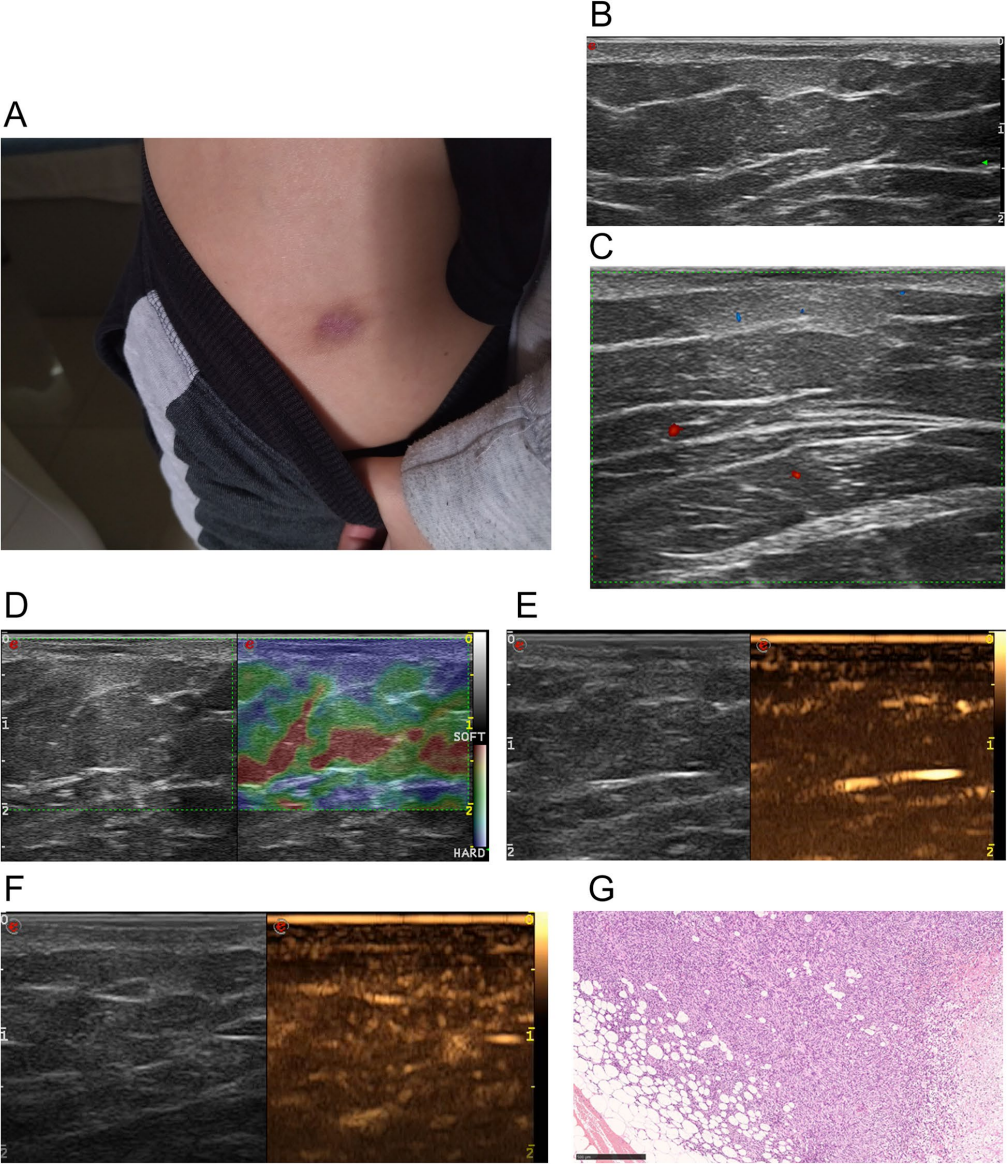
**A** DFSP with atrophic plague in the left thigh of a 40-year-old woman. **B** Transverse ultrasonogram revealing an ill-defined, inverted triangle hyperechoic subcutaneous with hypoechoic area in the sunken dermis, heterogeneous, and subcutaneous lesion. **C** Color Doppler ultrasonogram revealing intralesional vascularity and poor vascularization with vascular densities reaching 1.0/cm^2^. **D** Elastography showing an elasticity score of 4. **E** Contrast-enhanced ultrasound revealed the trend that contrast agent enters (the 5th second after the injection of contrast agent) the lesion from the bottom to the center. **F** Contrast-enhanced ultrasound revealed heterogeneous hyper-enhancement at peak (the 45th second after the injection of contrast agent), with the maximum diameter and depth of 2.15 cm and 1.1 cm respectively. **G** The hyperechoic area correlated with a honeycomb invasive pattern in the subcutaneous tissue on histology (hematoxylin-eosin × 40)

On power Doppler, DFSP lesions showed mainly artery of low velocity and low resistance index (RI). Eight DFSP lesions were measured using a 3-D superficial probe. 3-D color Doppler imaging revealed branch-shaped blood flow for 4 rich-vascular lesions, striped blood flow for 3 poor-vascular lesions, and wrapped blood flow for 1 rich-vascular lesion. Elastography was performed in 17 cases, 13 (76.5%) for which lesions were 4-score and 4 (23.5%) for which lesions were 3-score. Nineteen DFSP lesions were measured with CEUS. Ultrasound features are detailed in Tables 1 and 2. Representative ultrasonography findings from DFSP are shown in Figs. 1, 2, 3 and 4.

**Table 1:** Ultrasonography findings of DFSP

Indicator	DFSP
Cases, n	40
Depth (cm)	1.29 ± 0.79
The maximum diameter (n)	3.18 ± 1.31 (29)
Vascular density (/cm^2^)	2.1 ± 1.24
Venous blood flow velocity (cm/s) (n)	7.88 ± 5.76 (28)
Arterial PSV (cm/s) (n)	22.22 ± 11.98 (37)
Resistive index (n)	0.63 ± 0.1 (37)
Elasticity score (n)	3.78 ± 0.43 (17)
Heterogeneous echostructure, n (%)	34 (85)
Irregular margin, n (%)	26 (65)
Multilayer involvement	8 (20)
Ultrasound pattern, n (%)	
Hypoechoic	10 (25)
Hypoechoic with finger-like projections	4 (10)
Hypoechoic with finger-like projections and posterior hyperechoic area	12 (30)
Inverted triangle hyperechoic subcutaneous with hypoechoic area in the sunken dermis	4 (10)
Hyperechoic with a few hypoechoic bands	3 (7.5)
Mixed	7 (17.5)
Vascularity, n (%)	
No	2 (5)
Poor	17 (42.5)
Rich	21 (52.5)
Vascularity pattern, n (%)	
None	2 (5)
Peripheral	12 (30)
Intralesional	18 (45)
Peripheral and intralesional	8 (20)

Data had normal distribution and presented as mean ± SD, *PSV* peak systolic blood flow velocity

**Table 2:** Contrast-enhanced ultrasound findings of DFSP

Indicator	DFSP
Cases, n	19
Homogeneity, n (%)	
Homogeneous	5 (26.3)
Heterogeneous	14 (73.7)
Enhanced intensity, n (%)	
Iso-enhancement	2 (10.5)
Hyper-enhancement	17 (89.5)
TP (s)	41.4 ± 13.1
Peak	24.9 ± 17.2

*TP* peak time

DFSP depths and the maximum diameters assessed by US, CEUS and histology are shown in Table 3. CEUS showed better concordance than US with difference of just 0.01 cm on depth and 0.03 cm on the maximum diameter respectively compared with histology.

**Table 3:** Depth and the maximum diameter of DFSP by ultrasound, contrast-enhanced ultrasound and histology

Indicator	DFSP
Cases, n	19
US	
Depth (cm)	1.69 ± 1.12
The maximum diameter (cm)	3.51 ± 1.00
CEUS	
Depth (cm)	1.78 ± 1.11
The maximum diameter (cm)	3.64 ± 0.97
Histology	
Depth (cm)	1.79 ± 1.10
The maximum diameter (cm)	3.68 ± 1.01
Depth	
Difference (Histology and US, cm)	0.10 ± 0.14
Difference (Histology and CEUS, cm)	0.01 ± 0.09
P1	0.002
P2	0.000
The maximum diameter	
Difference (Histology and US, cm)	0.17 ± 0.16
Difference (Histology and CEUS, cm)	0.03 ± 0.16
P1	0.001
P2	0.043

Data had normal distribution and presented as mean ± SD, *US* ultrasound, *CEUS* contrast-enhanced ultrasound, *P*_*1*_ US and histology, *P*_*2*_ CEUS and histology

## Discussion

DFSP can be locally aggressive with a high recurrence rate, especially if it is not excised with negative margins [3]. Therefore, it is important to evaluate the extent of the tumor involvement. Examination of imaging studies are important for surgical planning. High frequency ultrasound can be used to evaluate the extent of the tumor involvement as well as provide surgery guidance. Ultrasound is increasingly being used in DFSP. To our knowledge, the present study is the largest series to describe multimodal ultrasound features of DFSP, and the first to investigate the usefulness of elastography and CEUS.

In our study, the DFSP patients mostly demonstrated red nodule and skin-colored dermal plaque, frequently located on the trunk and had equal distribution between males and females, and mostly occurs in young and middle-aged patients, which were in accordance with previous studies [4–7, 18, 19].

The gray-scale ultrasound findings of this 40-case series are mostly consistent with those described to date. Most of the tumors in our study appeared as hypoechoic lesions with occasional hyperechoic bands within the tumor matrix and lobulated lateral margins. More than half (26/40, 65%) of all DFSP lesions were irregular margin, which was correlated with an invasive growth pattern. Eight (20%) cases on ultrasound showed multilayer involvement, which was consistent with a locally aggressive growth pattern. Sixteen (40%) cases showed finger-like projections; such projections have been reported as a classic ultrasound feature in DFSP [14, 15, 18, 19]. Correlations have been previously reported between finger-like projections on ultrasound and radial or vertical wedge-shaped extensions of DFSP along the septa on histopathology [12, 14, 15, 18]. Cases showed a hyperechoic posterior area surrounding these projections, which correlated with the classic histopathological honeycomb pattern [18]. Cases with a mixed hyperechoic-hypoechoic pattern correlated with a multilayered histopathological growth pattern comprising bundles of spindle cells predominantly oriented parallel to the skin surface [2]; Cases with hyperechoic rings correlated with the compressive growth pattern [2]. Cases present as hyperechoic with a few hypoechoic bands correlated with tumor cells and fibrous tissues that are mixed together and infiltrating the surrounding subcutaneous fat tissue [8]. Previous study reported that one patient had one ultrasound pattern [18], however, we found that three kinds of ultrasound patterns could be presented on one patient, such as the case of Fig. 1.

There were four cases of ultrasound findings as inverted triangle hyperechoic subcutaneous with hypoechoic area in the sunken dermis, which was first reported in our study. Histologically, these cases were atrophic DFSP, a rare variant comprising only 1.7% of DFSP [22, 23]. The hypoechoic area also correlated with a classical pathognomonic storiform pattern, and the hyperechoic area correlated with fibrous tissues and small numbers of tumor cells [8, 22].

Cell density in DFSP is higher in the center of the tumor than at the edges, where finger-like projections containing small numbers of tumor cells can infiltrate tissue located far from the main tumor mass [24, 25]. Therefore, in our study the hypo-echo was mainly in the center of the mass, and the hyper-echo was mainly at the bottom or formed ring. One probable limitation of gray-scale ultrasound is underestimation of DFSP tiny extensions. Thus, we investigated the color Doppler patterns and found that 95% of lesions showed increased vascularity. This percentage is higher than the previously reported 85.7% [15], which revealed that increased vascularity supports an invasive growth pattern. On color Doppler, 52.5% of lesions were rich, which is higher than the previously reported 22.7% [19]; The hypo-echo was mainly in the center of the mass, which was consist with its intralesional vascular pattern. Furthermore, we found that many hyper-echo areas showed increased vascularity, which reflected some tiny extensions. Rich vascular lesions were primarily hypoechoic and mixed, which revealed that these two types of lesions had more blood vessels.

Regarding hemodynamics, our analysis revealed mainly artery of a moderate arterial peak systolic blood flow in DFSP, which reflects its markedly vascularized and invasive nature. A low RI reflected arteriovenous fistula, which prompted existing immature new blood vessels. On 3-D color Doppler ultrasound, DFSPs exhibited branch-shaped, striped, and wrapped color patterns, which was consist with intralesional, peripheral, and both vascular patterns. This feature supports DFSP as having moderate vascular density and an active nature.

The elasticity of a lesion can be used to differentiate it as benign or malignant, according to its score. This method of classification proved to be more accurate than palpation. Red, green, and blue colors represented soft, moderate, and hard, respectively. In our study, elastography showed the hypoechogenicity lesion area as mainly blue or green and the hyperechogenicity lesion area as primarily red. Accordingly, hypo-echo reflected tumor cells and hyper-echo reflected tumor and fat or fiber cells, revealing the more hypoechoic DFSP as more aggressive. Elastography demonstrated that DFSP was hard, further suggesting an aggressive nature consistent with its growth pattern.

Our study focused on the usefulness of CEUS as a preoperative planning tool for tumor resection. Unlike the conventional B-Mode ultrasound, CEUS uses contrast-enhanced agents such as SonoVue. Because tumor tissue has higher perfusion and thus, fills more rapidly with SonoVue compared to the surrounding normal tissue, CEUS outlines the border and blood flow perfusion of tumor tissue more precisely than the conventional ultrasound does. Ma C et al. [13] reported one DFSP case with CEUS of homogeneous iso-enhancement. In our 19-case series, 73.7% of lesions showed a heterogeneous contrast-enhancement and 89.5% of lesions showed hyper-enhancement, which reflects its malignancy. DFSPs showed long peak time and low peak, which is potentially related its presence as superficial or sometimes multi-layer-involved and not deep or extracutaneous.

CEUS is considered to be an effective technique to evaluate micro- vascularization. This is significant, because angiogenesis is the basis for neoplastic growth [26]. Previous one case report found that CEUS could improve precision of resection [13]. In our study, CEUS was more sensitive than color Doppler ultrasound to demonstrate blood perfusion, especially for peripheral vascularity. Nineteen DFSPs depth and the maximum diameter concordance between CEUS and histology were better than US and histology, with difference of only 0.01 cm and 0.03 cm respectively. This study showed that CEUS could be an effective pre-surgery inspection method for DFSP surgical treatment.

This study has a few limitations. First, elastography and CEUS applied only to a part of the population, according to this retrospective study we found that these two methods played important role for DFSP diagnosis, therefore in future work, we would continue to collect more cases of DFSP by CEUS and elastography. Second, the elastic scoring method was qualitative and lacked some objective basis. Third, there was insufficient correspondence between US and MRI features. Although the presence of DFSP could be suggested on US, it was difficult to evaluate its precise nature when the lesion was too large. Fourth, because the present case series constitutes a single-center study with a small sample size, multi-center studies and larger sample sizes are needed for continued research.

## Conclusion

We have presented what we believe to be the largest series of DFSP assessed by ultrasound to date. The results revealed that DFSP was more likely to be hypoechoic with occasional hyperechoic bands within the tumor matrix and lobulated lateral margins; marked by increased vascularity on 2-D ultrasound; characterized by branch-shaped, striped, and wrapped color patterns on 3-D color ultrasound; hard on elastography and heterogeneous hyper-enhanced on CEUS. Awareness of these multimodal US features should help doctors in diagnosis of DFSP. CEUS is a good tool to evaluate micro-vascularization, which could determine tiny tumor extension and preoperative margins.

## Data Availability

The datasets used and/or analyzed during the current study are available from the corresponding author on reasonable request.
